# Supplementary figures and images for: The ortholog of human REEP1-4 is required for autophagosomal enclosure of ER-phagy/nucleophagy cargos in fission yeast
Source: PLoS Biol. 2023 Nov 8;21(11):e3002372. doi: 10.1371/journal.pbio.3002372 (PMC10659188; doi:10.1371/journal.pbio.3002372)

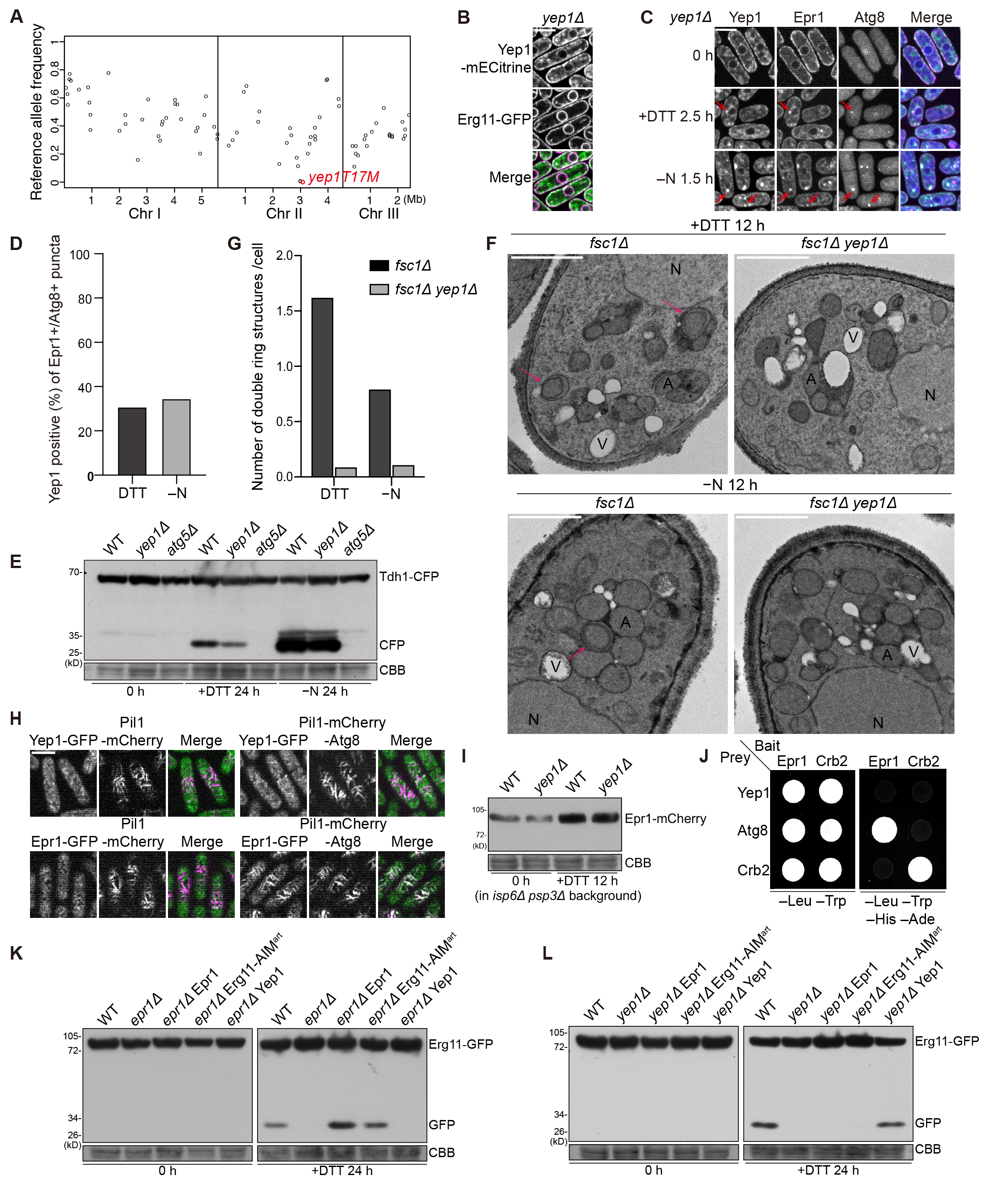

Supplement: S1 Fig — (A) Bulk segregant analysis identifying a mutation in SPBC30D10.09c (yep1) as a candidate phenotype-causing mutation in an ER-phagy defective mutant. The scatter plot depicts the reference allele frequencies at SNP sites in the pool of the ER-phagy defective segregants derived from a cross between the mutant strain and a wild-type strain. The T17M mutation in SPBC30D10.09c (yep1) is highlighted in red. (B) Subcellular localization of Yep1-mCherry expressed from the P41nmt1 promoter. Log-phase yep1Δ cells coexpressing Yep1-mCherry and the ER marker Erg11-GFP were examined by fluorescence microscopy. Bar, 5 μm. (C) Yep1-mECitrine formed puncta colocalizing with Epr1 and Atg8 double positive puncta after ER-phagy induction by nitrogen starvation and DTT treatment. Red arrows denote puncta where Yep1-mECitrine, Epr1-mCherry, and mTurquoise2-Atg8 colocalize. Bar, 5 μm. (D) Quantification of the percentage of Epr1 and Atg8 double positive (Atg8+/Epr1+) puncta that are also positive for Yep1 in the analysis shown in (C) (more than 100 Atg8+/Epr1+ puncta were examined for each sample). (E) Autophagic processing of the bulk autophagy marker Tdh1-CFP was largely normal in yep1Δ cells. (F) Electron microscopy analysis of starved and DTT-treated fsc1Δ and fsc1Δ yep1Δ cells. N, nucleus; V, vacuole; A, autophagosome. Double-ring structures are denoted by pink arrows. Bar, 1 μm. (G) Quantification of the number of double-ring structures per cell in the analysis shown in (E) (more than 50 cells with autophagosomes were examined for each sample). (H) Yep1 did not interact with Atg8 in a Pil1 co-tethering assay. Log-phase cells coexpressing the bait (Pil1-mCherry or Pil1-mCherry-Atg8) and the prey Yep1-GFP were examined by fluorescence microscopy. Cells coexpressing Pil1-mCherry-Atg8 and Epr1-GFP served as a positive control. Peripheral planes of the cells were imaged. Bar, 5 μm. (I) Yep1 is not required for the DTT-induced increase of the protein level of Epr1. isp6Δ psp3Δ backgroun [file pbio.3002372.s001.tif]

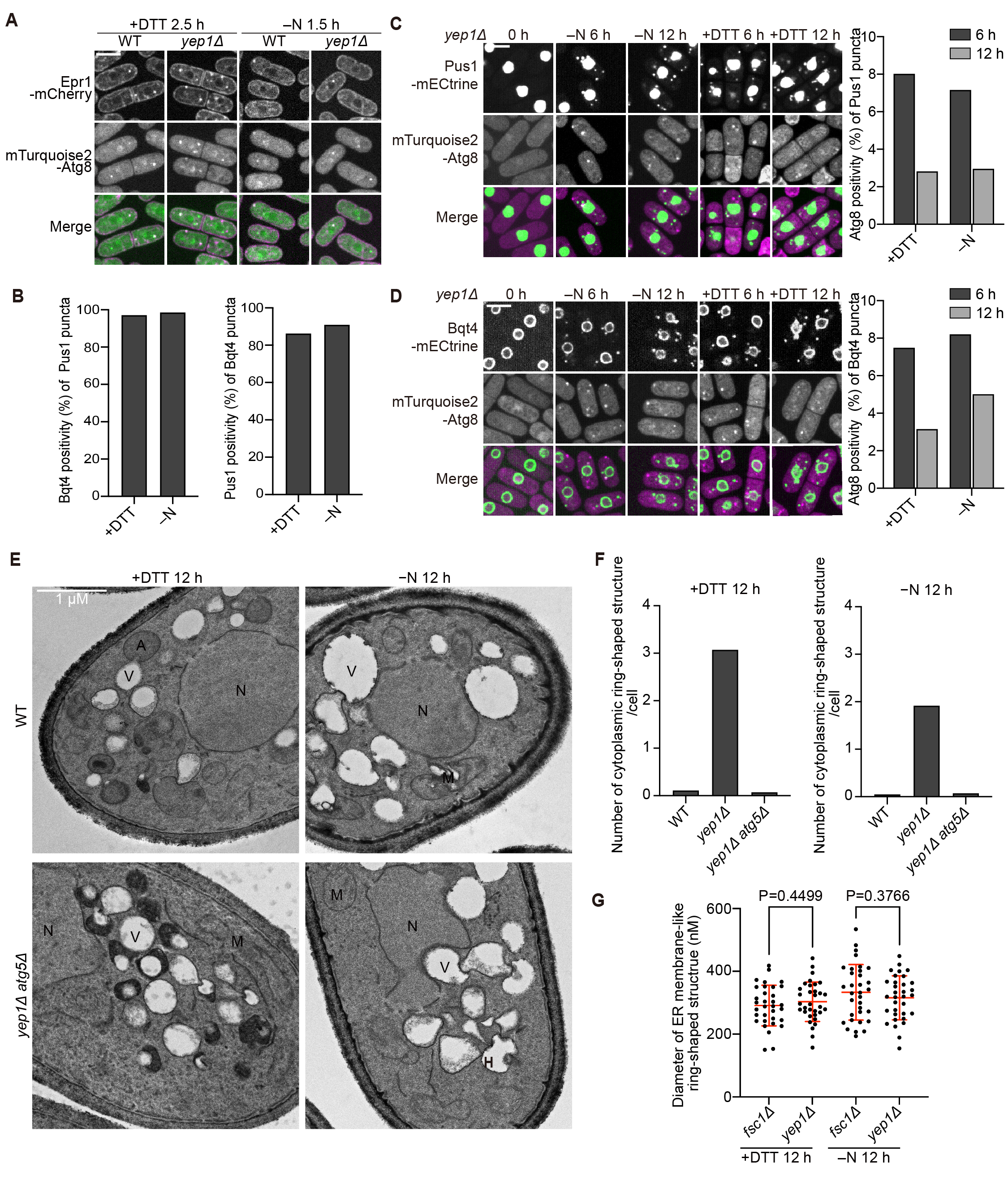

Supplement: S2 Fig — (A) Yep1 is not required for the colocalization of Epr1 and Atg8 at punctate structures shortly after ER-phagy induction. Wild-type and yep1Δ cells coexpressing Epr1-mCherry and mTurquoise2-Atg8 were examined by microscopy after 2.5-hour DTT or 1.5-hour starvation treatment. Bar, 5 μm. (B) Quantification of the colocalization between Bqt4 puncta and Pus1 puncta in the analysis shown in Fig 2C (more than 250 puncta were examined for each sample). (C, D) The vast majority of cytoplasmic Pus1 puncta (C) and Bqt4 puncta (D) in yep1Δ cells did not colocalize with Atg8 puncta. Bar, 5 μm. Over 200 Pus1 or Bqt4 puncta were examined per sample. (E) Electron microscopy analysis of nitrogen-starved and DTT-treated wild-type and yep1Δ atg5Δ cells. N, nucleus; V, vacuole; M, mitochondrion; A, autophagosome. Bar, 1 μm. (F) Quantification of the number of cytoplasmic ring-shaped structures in the analysis shown in Figs 2D and S2E (more than 30 cells were examined for each sample). Autophagosomes, which are ring-shaped structures juxtaposed to vacuoles, were excluded from this quantification. (G) Quantification of the diameters of the ring-shaped structures in yep1Δ cells in the analysis shown in Fig 2D and the inner rings in the double-ring structures in fsc1Δ cells in the analysis shown in S1F Fig. P values were calculated using Welch’s t test. Numerical data underlying panels B-D, F, and G can be found in S1 Data. (TIF) [file pbio.3002372.s002.tif]

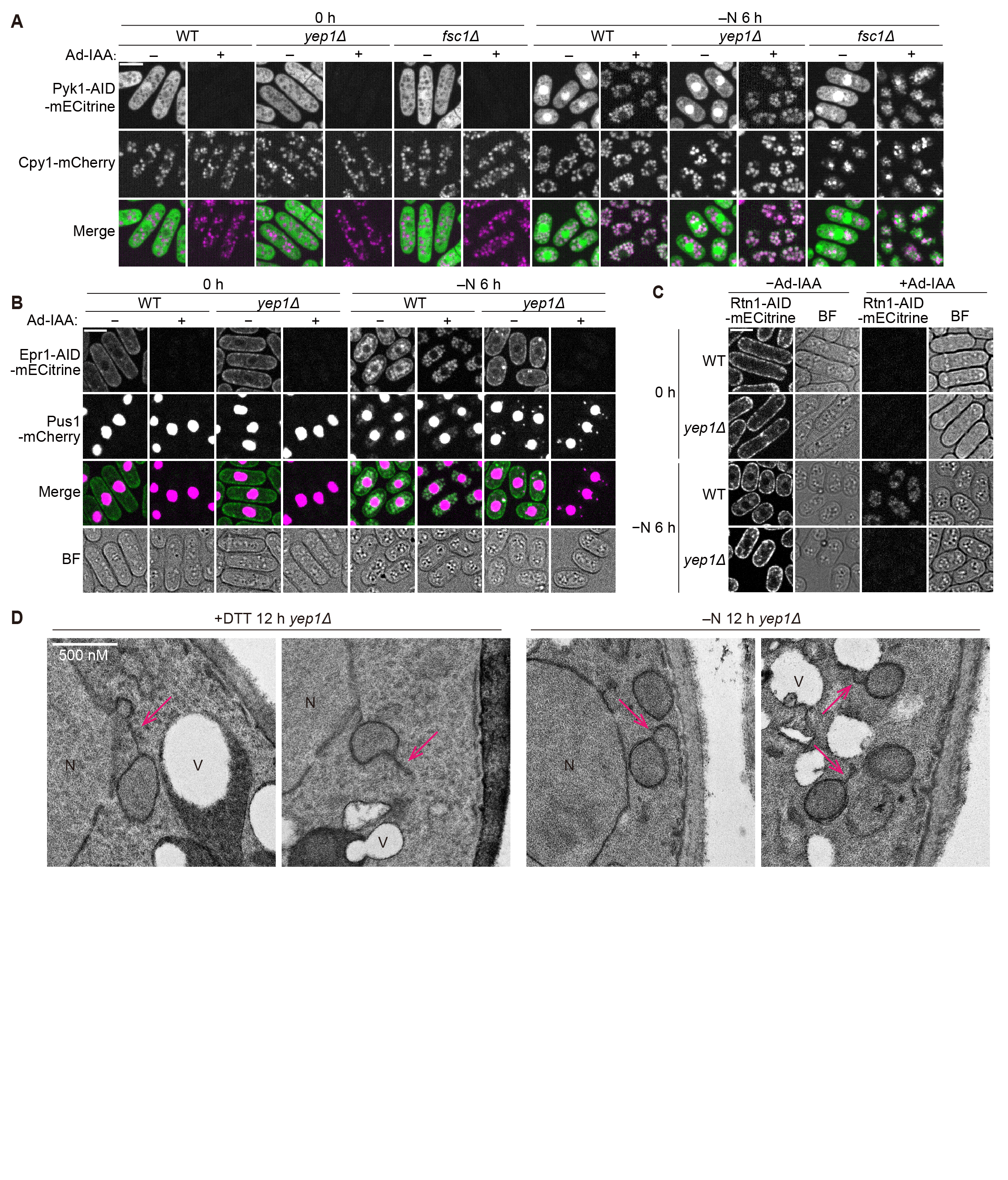

Supplement: S3 Fig — (A) Applying the degron protection assay on the cytosolic protein Pyk1-AID-mECitrine. Prior to observation, cells were treated with (+Ad-IAA) or without (−Ad-IAA) 5-adamantyl-IAA for 1.5 hours. BF, brightfield. Cpy1-mCherry is a vacuole lumen marker. Bar, 5 μm. (B) Applying the degron protection assay on Epr1-AID-mECitrine. BF, brightfield. Bar, 5 μm. (C) Applying the degron protection assay on Rtn1-AID-mECitrine. BF, brightfield. Bar, 5 μm. (D) Electron microscopy images of ER-phagy/nucleophagy cargo structures with filamentous membrane protrusions in yep1Δ cells. N, nucleus; V, vacuole. Pink arrows denote filamentous membrane protrusions that extend from the ER-phagy/nucleophagy cargo structures. Bar, 1 μm. (TIF) [file pbio.3002372.s003.tif]

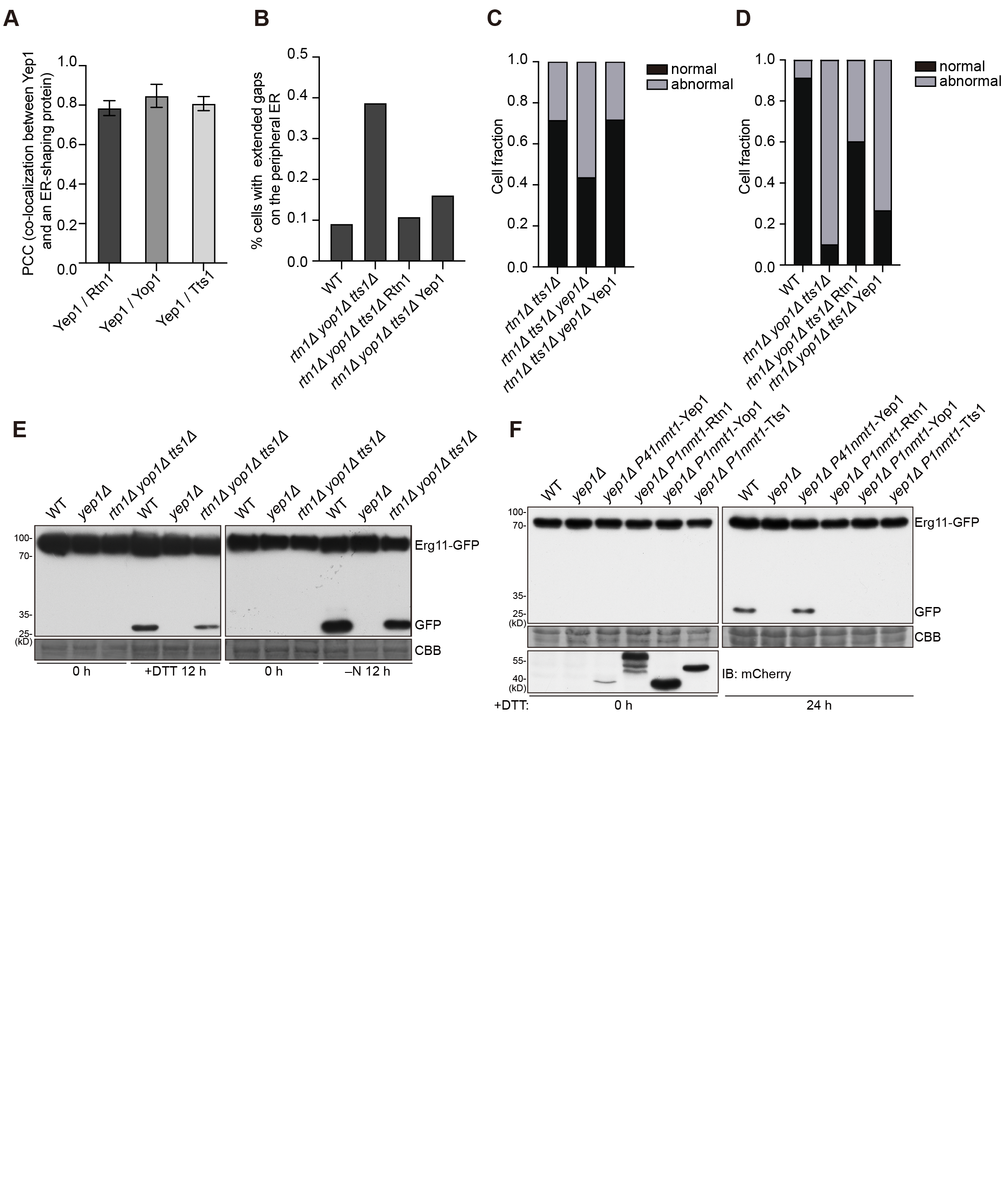

Supplement: S4 Fig — (A) Colocalization between Yep1-mCherry and Rtn1-GFP, Yop1-GFP, or Tts1-GFP was quantitated using Pearson’s correlation coefficient (PCC). The PCC values are presented as mean ± SD (n = 10 cells). (B) Quantification of the percentages of cells with extended gaps in images of the midplane in the analysis shown in Fig 3B (more than 300 cells were examined for each sample). (C, D) Quantification of the septum abnormality phenotypes (more than 200 cells with septa were examined for each sample). (E) ER-phagy induced by DTT treatment or nitrogen starvation was largely normal in the absence of Rtn1, Yop1, and Tts1. (F) The ER-phagy defect of yep1Δ cells was not suppressed by the ectopic expression of Rtn1, Yop1, or Tts1. Proteins expressed in yep1Δ were tagged with mCherry, and their expression levels were analyzed by immunoblotting using an antibody against mCherry. Numerical data underlying panels A-D can be found in S1 Data, and raw images for panels E and F can be found in S1 Raw Images. (TIF) [file pbio.3002372.s004.tif]

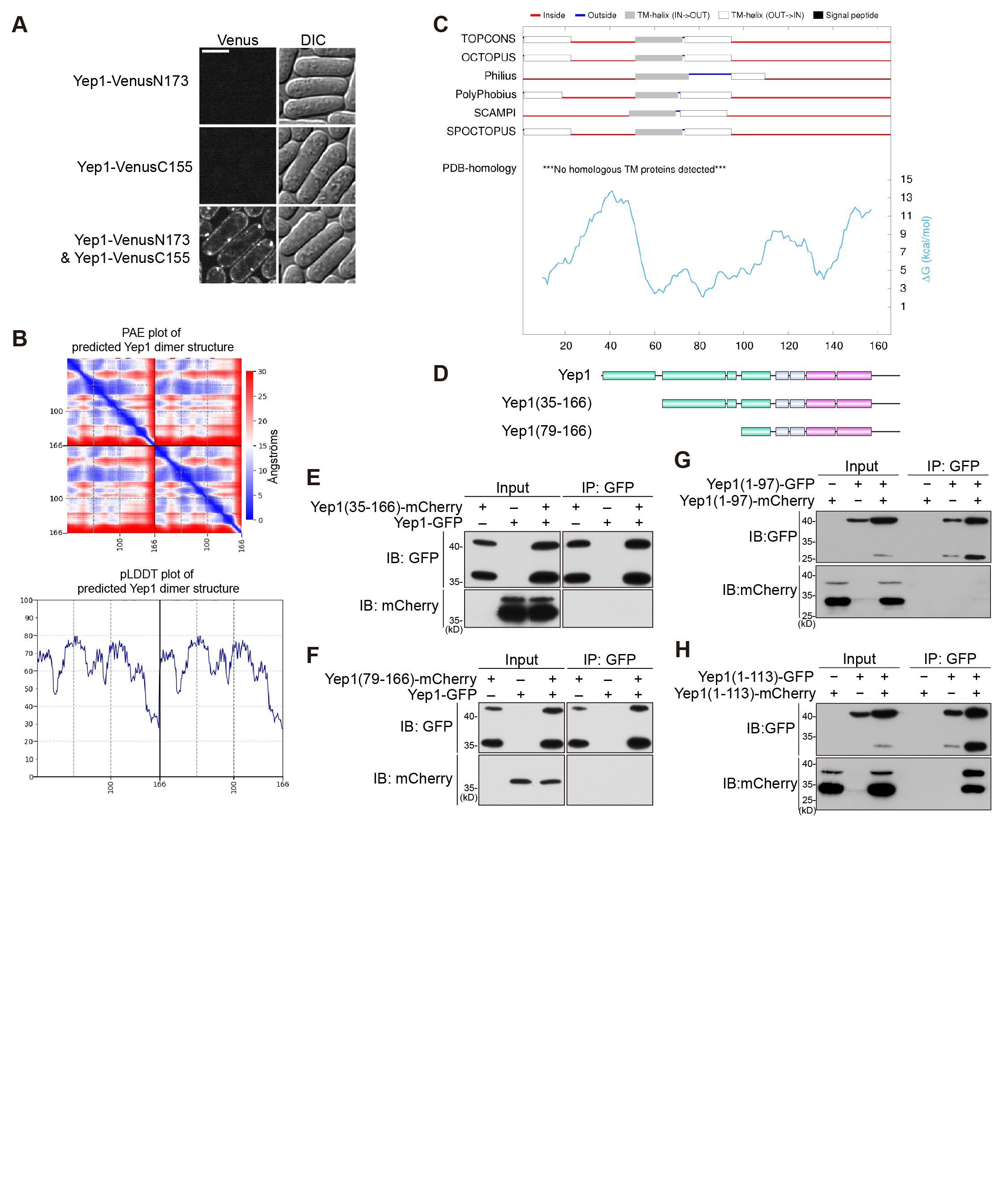

Supplement: S5 Fig — (A) Yep1 exhibited self-interaction in the BiFC assay. Log-phase cells expressing Yep1-VenusN173 alone, Yep1-VenusC155 alone, or both were examined by fluorescence microscopy. Bar, 5 μm. (B) The predicted aligned error (PAE) plot and pLDDT plot of the AlphaFold-Multimer-predicted structure of the Yep1 homodimer shown in Fig 3F. (C) TOPCONS membrane protein topology prediction for Yep1. (D) Schematics of wild-type and truncated Yep1. (E) Yep1 (35–166) did not interact with full-length Yep1 in a co-immunoprecipitation analysis. (F) Yep1 (79–166) did not interact with full-length Yep1 in a co-immunoprecipitation analysis. (G) Yep1 (1–97) did not exhibit self-interaction in a co-immunoprecipitation analysis. (H) Yep1 (1–113) exhibited self-interaction in a co-immunoprecipitation analysis. Numerical data underlying panels B and C can be found in S1 Data, and raw images for panel E-H can be found in S1 Raw Images. (TIF) [file pbio.3002372.s005.tif]

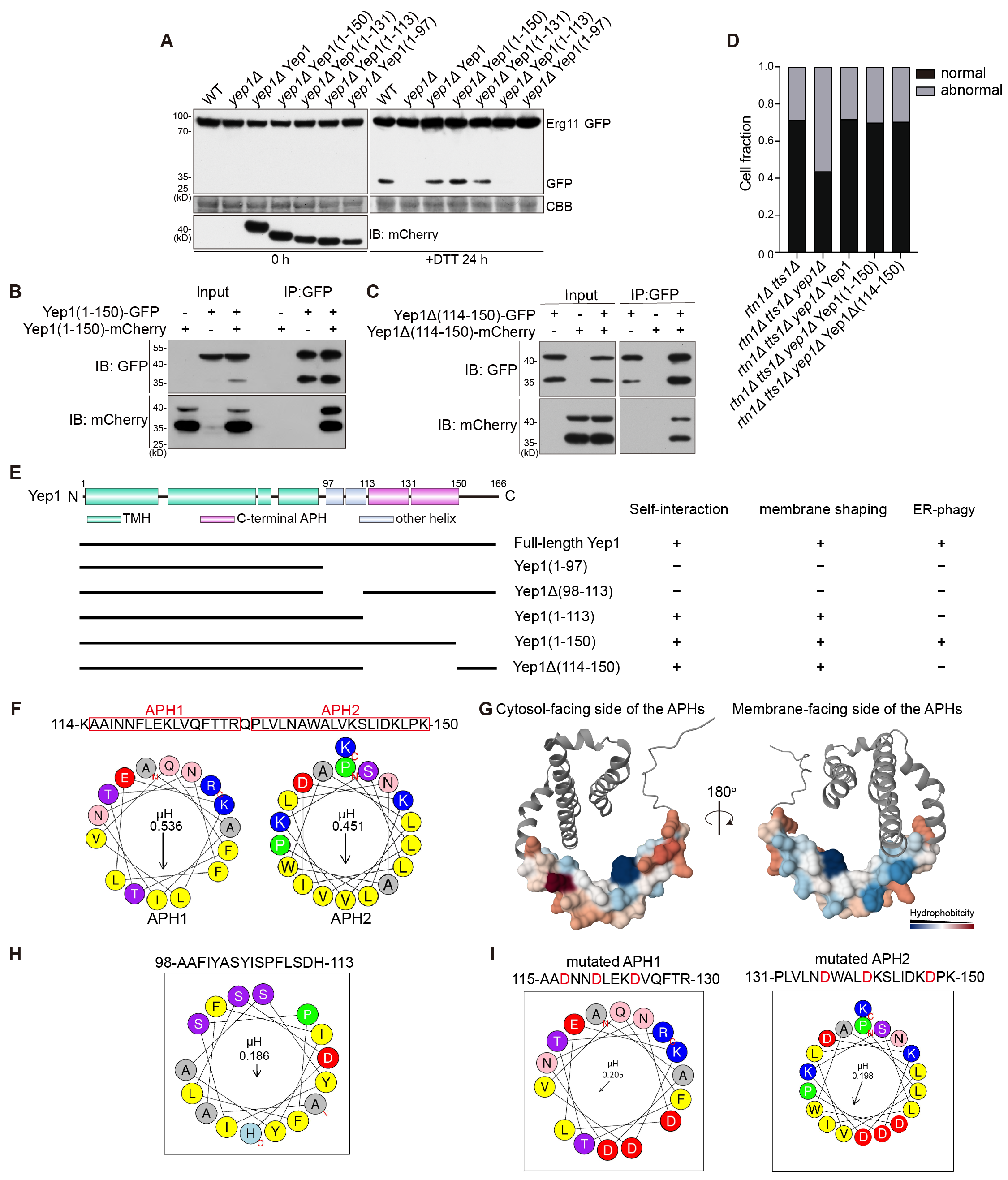

Supplement: S6 Fig — (A) Yep1 (1–131) or Yep1 (1–150), but not Yep1(1–113), is able to support ER-phagy. (B) Yep1 (1–150) exhibited self-interaction in a co-immunoprecipitation analysis. (C) Yep1Δ (114–150) exhibited self-interaction in a co-immunoprecipitation analysis. (D) Quantification of the septum abnormality phenotypes in rtn1Δ tts1Δ cells, rtn1Δ tts1Δ yep1Δ cells, and rtn1Δ tts1Δ yep1Δ cells expressing full-length Yep1, Yep1 (1–150), or Yep1Δ (114–150) (more than 200 cells with septa were examined for each sample). (E) Summary of the truncation and internal deletion analysis of Yep1. (F) Helical wheel representations of 2 APHs of Yep1. The helical wheels were generated using HeliQuest. Hydrophobic residues are colored in yellow, hydrophilic residues in blue (R and K), red (D and E), purple (T and S), and pink (N and Q), alanine in grey, and proline in green. The HeliQuest-calculated hydrophobic moment (μH) of the helix is shown. (G) The amphipathic nature of APHs is visualized in the AlphaFold-Multimer-predicted structure. The 2 APHs and the intervening amino acid are shown in the surface representation and are colored base on hydrophobicity. The rest of Yep1 is shown in the cartoon representation. (H) Helical wheel representation and the hydrophobic moment (μH) of residues 97–113 of Yep1. (I) Helical wheel representations and the hydrophobic moments (μH) of mutated APHs. Numerical data underlying panel D can be found in S1 Data, and raw images for panels A-C can be found in S1 Raw Images. (TIF) [file pbio.3002372.s006.tif]

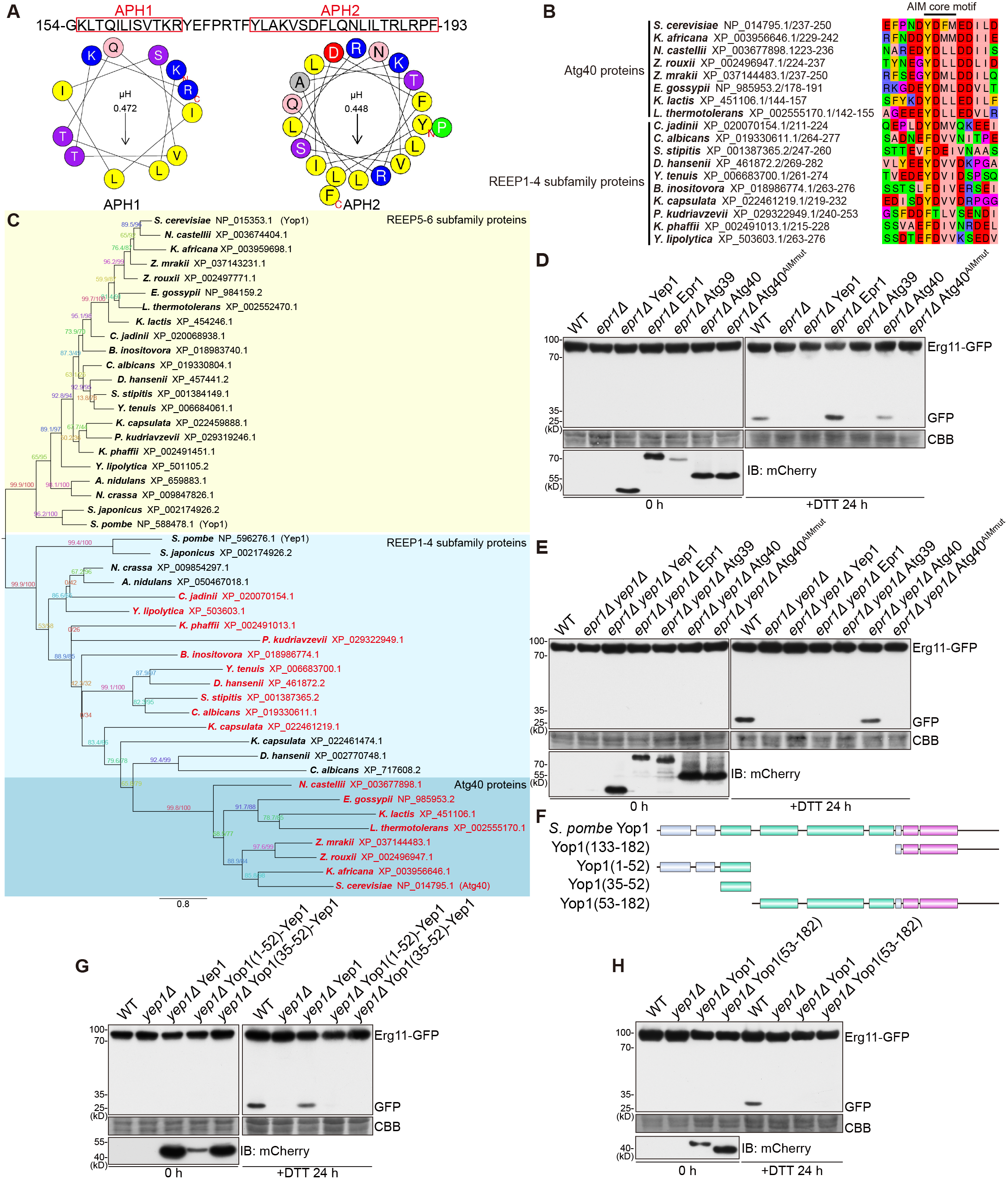

Supplement: S7 Fig — (A) Helical wheel representations and the hydrophobic moments (μH) of the 2 C-terminal APHs in Atg40. (B) The alignment of the C-terminal AIM in the proteins whose names are colored red in (C). The AIM core motif is highlighted. (C) Phylogenetic relationships of REEP family proteins and Atg40 proteins in representative Ascomycota species. The sequences of REEP family proteins were retrieved by PSI–BLAST from the NCBI refseq_protein database using the sequences of Yarrowia lipolytica orthologs of S. pombe Yop1 and Yep1 as queries. The sequences of Atg40 proteins were retrieved by PSI–BLAST from the NCBI refseq_protein database using the sequence of S. cerevisiae Atg40 as query. A sequence alignment was generated using MAFFT, and a maximum likelihood tree was constructed using IQ-TREE. The tree was rooted using the REEP5-6 subfamily proteins as outgroup. Branch labels are the SH-aLRT support values (%) and the UFBoot support values (%) calculated by IQ-TREE. The names of proteins containing a C-terminal AIM are colored red. The scale bar indicates 0.8 substitutions per site. (D) S. cerevisiae Atg40 rescued the ER-phagy defect of epr1Δ in a manner dependent on its Atg8-interacting motif (AIM). Atg40AIMmut harbors the Y242A and M245A mutations. Proteins expressed in yep1Δ were tagged with mCherry, and their expression levels were analyzed by immunoblotting using an antibody against mCherry. (E) S. cerevisiae Atg40 rescued the ER-phagy defect of epr1Δ yep1Δ in a manner dependent on its AIM. Proteins expressed in yep1Δ were tagged with mCherry, and their expression levels were analyzed by immunoblotting using an antibody against mCherry. (F) Schematics of wild-type and truncated Yop1. Yop1 (133–182) appears in Fig 4I. Yop1 [1–52] and Yop1 [35–52] appear in (G). Yop1 (53–182) appears in (H). (G) Adding an extra N-terminal transmembrane helix (TMH) to Yep1 disrupted its ER-phagy function. Yop1 [1–52] includes the N-terminal cytosolic region and the first TMH of Yop1. Yop1 [ [file pbio.3002372.s007.tif]

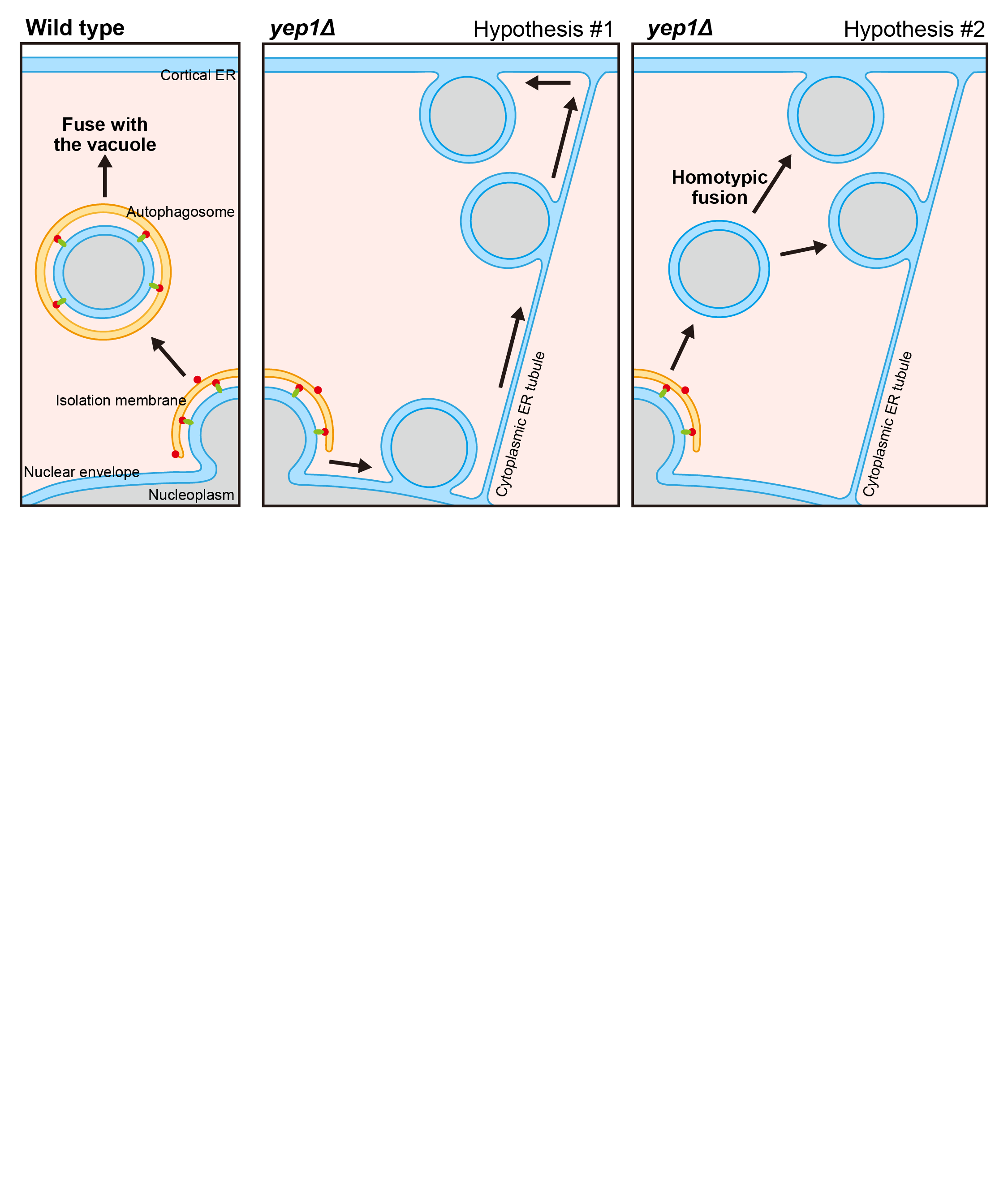

Supplement: S8 Fig — In wild-type cells, ER-phagy/nucleophagy cargos are sequestered into autophagosomes after their separation from the source compartments and are delivered to the vacuole through autophagosome–vacuole fusion. In the absence of Yep1, the recruitment of the autophagic machinery at the early phase of ER-phagy/nucleophagy occurs normally, but ER-phagy/nucleophagy cargos fail to be delivered to the vacuole. Instead, ER-phagy/nucleophagy cargo structures not enclosed within autophagosomes accumulate in the cytoplasm. The outer membranes of these structures remain continuous with the nuclear envelope-ER network. In hypothesis 1, we propose that fission of the outer membranes of ER-phagy/nucleophagy cargos fails to occur during cargo separation, resulting in the formation of luminal vesicles. These vesicles may move along the cytoplasmic ER tubules. In hypothesis 2, cargo separation happens but autophagosome enclosure somehow fails. Fully separated cargos reassociate with the ER network through homotypic fusion. (TIF) [file pbio.3002372.s008.tif]

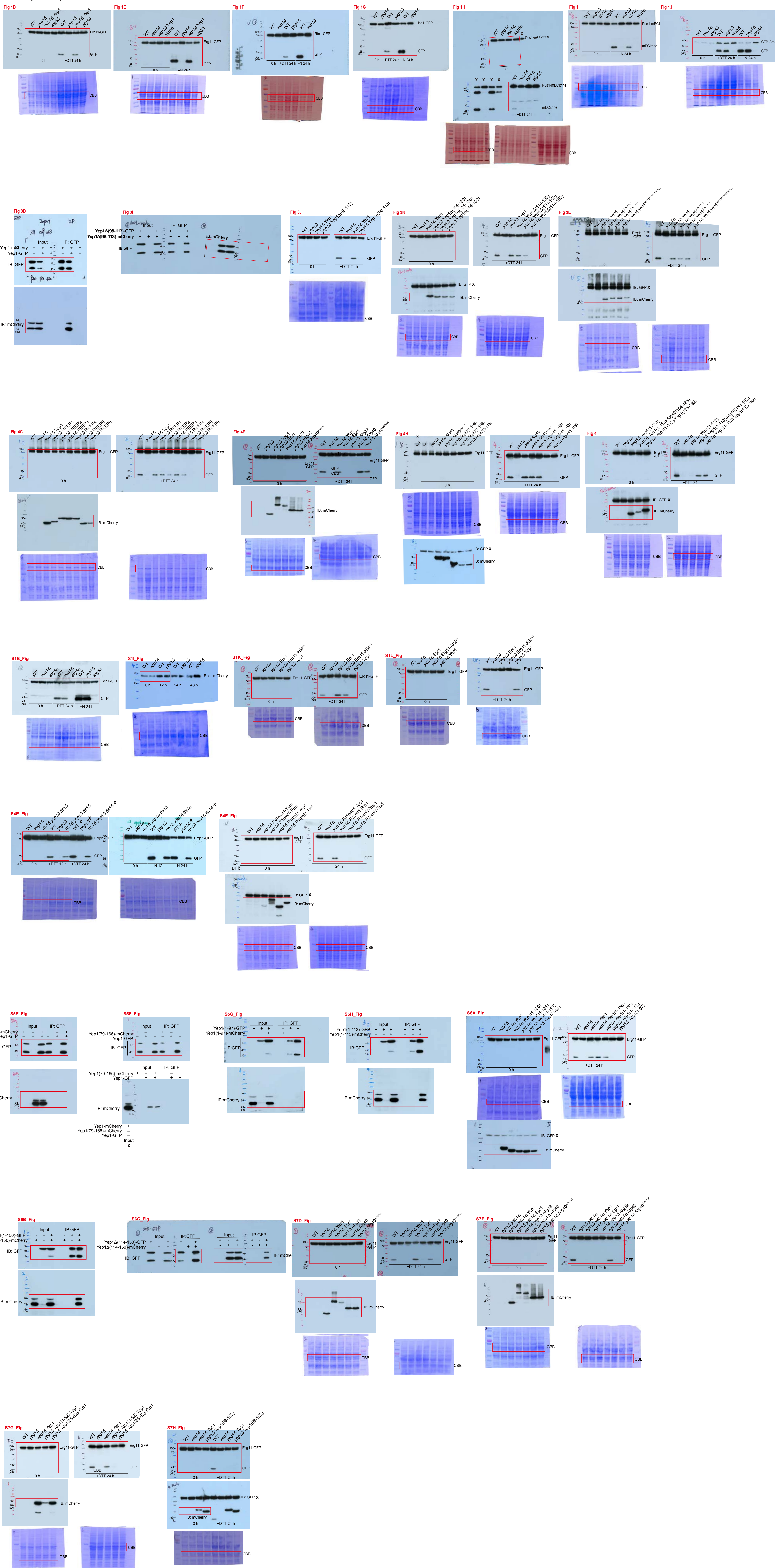

Supplement: S1 Raw Images — (PDF) [file pbio.3002372.s013.pdf]
