# Supplementary material for: The ortholog of human REEP1-4 is required for autophagosomal enclosure of ER-phagy/nucleophagy cargos in fission yeast
Source: PLoS Biol. 2023 Nov 8;21(11):e3002372. doi: 10.1371/journal.pbio.3002372 (PMC10659188; doi:10.1371/journal.pbio.3002372)
Supplement: Attachment — Submitted filename: Response to Reviewers-20230910-2.docx [file pbio.3002372.s014.docx]

Reviewer #1 (Noboru Mizushima, signs review): The authors conducted an imaging-based screen to identify ER-phagy factors and discovered a REEP-like protein named Yep1. Yep1 plays a specific role in ER-phagy and nucleophagy at the sequestration step but not the initiation step. Yep1 exhibits membrane-shaping activity and requires its oligomerization property and the C-terminal amphipathic helices for ER-phagy. Structurally similar mammalian REEP proteins and the budding yeast Atg40 (even lacking the AIM motif) can rescue the loss of Yep1. Atg40 in budding yeast is, therefore, like a hybrid molecule of Yep1 and Epr1 in fission yeast.

This study significantly contributes to our understanding of the mechanism of ER-phagy in fission yeast. It also provides a clear explanation for the lack of membrane-shaping activity in the previously known ER-phagy receptor Epr1. The data presented are well-organized and compelling, and this paper is worth publishing. There are only a few minor comments to address.

1. The authors discuss two possibilities regarding the function of Yep1: (1) shaping of the cargo membrane and/or (2) shaping of the autophagosomal membrane. While it is the authors' prerogative to consider both possibilities, this reviewer finds the first hypothesis to be much more likely based on the findings in the present study. It might be beneficial for the authors to provide a bit stronger suggestion for the first hypothesis in the Abstract. Again, the decision is up to the authors.

**Response: We thank the reviewer for the positive evaluation of our manuscript and for the insightful suggestions.** **We agree with the reviewer that the first hypothesis is more likely. Based on new experimental data generated during revision (see the third response to point 2 below), we have further** **refined this hypothesis (shown in Figure S8) and provided stronger suggestion for it in the abstract.**

2. The structures observed in DDT-treated yep1Δ cells are intriguing (Fig. 2B). However, it remains unclear why the formation of these structures is dependent on Atg5. This issue is not explained in the model presented in Fig. S7. It would be helpful to provide further explanation in the Discussion regarding ATG dependency.

**Response:** **In the** **Discussion section of the revised manuscript, we now speculate that the formation of these structures, which are cargos of ER-phagy and nucleophagy, may depend on the Atg machinery-mediated formation of the isolation membrane at the sites of ER-phagy and nucleophagy. It is plausible that the isolation membrane may be involved in providing a force for the budding of cargo membrane structures.**

Furthermore, there are some related questions. Are the cargo structures that accumulate in yep1Δ cells larger than those observed in autophagosomes in wild-type cells?

**Response: We quantified the diameters of the cargo** **structures that accumulate in the cytoplasm of** ***yep1Δ* cells and compared them to the diameters of cargo structures in autophagosomes in** ***fsc1Δ* cells, which** **are defective in autophagosome-vacuole fusion. The results are shown in Figure S2G. We found no significant difference in the diameters of these two types of structures.**

Are these structures completely detached from the source membranes (no minor connections)? Discussing these points would be helpful for readers.

**Response:** **To address the reviewer’s question, we have further analyzed the nucleophagy cargo structures that accumulate in the cytoplasm of *yep1Δ* cells during the revision. New data indicate that while the inner contents and the inner membranes of these structures are disconnected from the nucleus, the outer membranes remain continuous with the nuclear envelope/ER network. These results are presented in Figures 2F-2J and Figure S3D of the revised manuscript. Based on these new findings, we now hypothesize that** **Yep1 may be required for the fission of the outer membranes of ER-phagy/nucleophagy cargos. However, we cannot exclude the alternative possibility that cargo structures initially separate fully from the source compartments but later** **re-associate with the nuclear envelope/ER network through homotypic fusion. We discuss both of these possibilities in the Discussion section (Figure S8).**

3. Recently, a highly relevant paper was published by Kanki's group (PMID: 37191320). Please cite and discuss this paper.

**Response: In the revised manuscript, we now cite** **Fukuda et al. (PMID: 37191320) and discuss it in the Discussion section.**

Reviewer #2: In the manuscript, the authors identify the protein Yep1, the yeast ortholog of mammalian REEPs, as a component of the ER-phagy/nucleophagy machinery. Yep1 is not affecting the initiation of the autophagic process but is rather involved in the enclosure of the ER-phagy and nucleophagy cargos.

The ideas are interesting and the role of REEP proteins in autophagosome shaping and ER-phagy have been reported also by other groups (PMID: 37191320; doi.org/10.1101/2022.10.27.514035). The fact that two additional publications reported similar results is not really affecting the novelty. One paper has been published very recently and the other one is deposited in bioRxiv.

The main point with the manuscript is a lack of experimental context. The manuscript a bit confusing, the final message, that the authors want to deliver, is not clear. While the role of Yep1 is presented as crucial for cargos delivery to vacuole, on the other side the Atg40 protein seems to fully compensate for the absence of Yep1. Therefore, it is not clear the relation between Atg40 and Yep1. Despite the structural similarities regarding the amphipathic helixes, the two proteins are distinct. Further investigations are needed to establish the clear relation between the ER-phagy receptor Atg40 and ER structural protein Yep1. Do they directly interact? Are they involved in the formation of some protein clusters that functionally connect these proteins during the ER-phagy process?

**Response: Yep1 is an *S. pombe* REEP1-4 subfamily protein and Atg40 is an *S. cerevisiae* protein not sharing obvious sequence similarity with REEP1-4 subfamily proteins. During the revision of this manuscript, we have conducted a synteny analysis and found that the *S. cerevisiae* gene encoding Atg40 shares synteny with non-*S. cerevisiae* genes encoding REEP1-4 subfamily proteins. This new result is presented in Figure 4E of the revised manuscript. Based on this evidence and other data shown in Figure 4D and Figure S7C, we now conclude that Atg40 is an ortholog of Yep1 and other REEP1-4 subfamily proteins.**

**In *S. pombe*, the only known ER-phagy receptor is Epr1. We show in the manuscript that heterologous expression of *S. cerevisiae* Atg40 can rescue the ER-phagy defect of the *S. pombe* double deletion mutant lacking both Epr1 and Yep1. This finding suggests that *S. cerevisiae* Atg40 is functionally equivalent to the combination of *S. pombe* Epr1 and Yep1. Our evolutionary analysis presented in Figure 4D indicates that the common ancestor of the *Saccharomycotina* subphylum may have possessed the first REEP1-4 subfamily protein capable of serving dual roles of an ER-shaping protein and an Atg8-binding ER-phagy receptor.**

**To address the reviewer’s question on the relationship between Yep1 and the ER-phagy receptor, we have performed additional experiments during the revision. A yeast two-hybrid experiment showed that Yep1 does not interact directly with Epr1. The result of this experiment is presented in Figure S1J of the revised manuscript. In addition, we have performed an** **immunoprecipitation coupled with mass spectrometry (IP-MS) experiment using Yep1-GFP as bait. Epr1 was not co-immunoprecipitated with Yep1. However, a large number of ER membrane proteins were co-immunoprecipitated with Yep1. These co-immunoprecipitated ER proteins are listed in Table S1 of the revised manuscript. Among them are the two VAP-family proteins (Scs2 and Scs22) responsible for localizing Epr1 to the ER membrane (Zhao et al. 2019 PMID: 32735772). It is possible that VAPs may connect Yep1 to Epr1. We discuss these potential Yep1 interactions in the Discussion section. Further studies will be needed to elucidate their functional relevance.**

Moreover, many experiments are redundant and recapitulate already published data (all the experiments made with Rtn1 mutants are not really necessary and already reported in literature).

**Response: Zhang et al. 2010 (PMID: 20434336) have shown that three *S. pombe* ER-shaping proteins Rtn1, Yop1, and Tts1 act in a partially redundant manner to maintain tubular ER. However, the role of Yep1 in ER shape maintenance has not been investigated previously. Our analysis of ER shape-related phenotypes showed that overexpressing Yep1 can suppress the ER shape defect of the triple deletion mutant lacking Rtn1, Yop1, and Tts1, and *yep1Δ* caused a weak defect by itself and substantially enhanced the phenotypes of mutants lacking other ER-shaping proteins. These results not only reveal a role of Yep1 in maintaining ER shape, but also provide an in vivo assay for examining the ER-shaping activity of Yep1. Using this assay, we show that Yep1 self-interaction is important for its ER-shaping ability.**

Most of the experiments are based on yeast genetics, that is good, but the molecular mechanisms are also quite speculative. How Yep1 function is regulated? Is it part of a larger protein complex? Is it post-translationally modified?

**Response: Our unpublished immunoblotting results have not revealed evidence that the function of Yep1 is regulated. Specifically, we did not observe an increase in the protein level of Yep1 when ER phagy was induced by nitrogen starvation or DTT treatment. Additionally, immunoblotting analysis did not detect any shifted Yep1 bands that would indicate post-translational modifications of this protein.**

**Our newly performed IP-MS analysis using Yep1-GFP as bait enriched not only Scs2 and Scs22, as mentioned above, but also several ER shaping-proteins including Rtn1, Yop1, Tts1, Sey1 (atlastin homolog), and Lnp1 (lunapark homolog) (Table S1 in the revised manuscript). In the revised manuscript, we discuss the possibility that Yep1 may cooperate with other ER-shaping proteins during ER-phagy/nucleophagy, analogous to the situation in mammalian cells where FAM134B clusters with other ER-shaping proteins to drive ER-phagy (PMID: 37225996 and PMID: 37225994). Further studies will be needed to elucidate the precise mechanisms by which Yep1 and other ER-shaping proteins collaborate during autophagic processes.**

This manuscript has a potentially solid idea, but it needs quite a lot of improvements regarding the molecular mechanisms that are needed to support authors' hypothesis.

**Response: We agree with the reviewer that the molecular mechanisms underlying the role of Yep1 in ER-phagy/nucleophagy remain largely elusive. Further studies are warranted to elucidate the precise molecular mechanisms by which Yep1 promotes ER-phagy/nucleophagy.**

Authors should also consider including in the manuscript, maybe in the discussion the recent published literature on ER-phagy.

**Response: We have cited several recently published papers on ER-phagy in the** **revised manuscript.**

Reviewer #3: In this study, Zou et al. identified that the REEP family protein Yep1 as a novel factor involved in ER-phagy and nucleophagy in Schizosaccharomyces pombe. In cells lacking Yep1, ER-phagy and nucleophagy are completely blocked, but bulk autophagy is almost normal. Yep1 is not required for the initial step of ER-phagy in which the ER-phagy receptor Epr1 and Atg8 are colocalized. Based on the results of fluorescence microscopy and electron microscopy with the genetically encoded EM tag MTn, ring-shaped membrane structures derived from nucleus or ER were shown to accumulate in yep1Δ cells. Yep1, like known ER-shaping proteins such as Yop1 and Rtn1, has the ability to self-interact and promote ER morphogenesis. The authors showed that self-interaction and the C-terminal amphipathic helices of Yep1 are required for ER-phagy. The authors also showed that human REEP1-4 and budding yeast Atg40 can rescue the defect of ER-phagy in yep1Δ cells. Given these results, the authors propose that Yep1 functions in the autophagosomal enclosure of nucleus/ER derived membrane structures. The finding in this article is novel and important for understanding how the substrates in the ER and nucleus are sequestered into autophagosomes, but the authors need to address the following issues to improve the manuscript.

1. In Figure 1H and I, the authors stated that the deletion of EPR1 affected the degradation of Pus1-mCitrine in DTT-treated cells more severely than that in nitrogen starved cells. However, it seems that cleaved mECitrine was not detected in epr1Δ cells just because it was less produced even in wild-type cells treated with DTT rather than because ERP1 deletion more strongly impaired nucleophagy induced by DTT treatment.

**Response:** **We thank the reviewer for the positive evaluation of our manuscript and for providing constructive suggestions.** **We agree with the reviewer that Pus1-mECitrine processing was less pronounced under DTT treatment compared to starvation conditions in wild-type cells, making it difficult to compare the severity of the defect of the mutant under these two conditions. We have revised the manuscript and removed the claim that the defect is more severe under DTT treatment.**

2. When ER-phagy and nucleophagy are induced, does Yep1 accumulate in the site of the ER/nucleus derived vesicles formation? The authors only show the localization of Yep1 during the logarithmic growth phase. The authors should observe the localization of Yep1 when ER-phagy and nucleophagy are induced.

**Response: We have added new data (in Figures S1C and S1D) showing that when** **ER-phagy/nucleophagy was induced, Yep1 was observed to form colocalizing puncta at about 30% of Atg8 and Epr1 double positive puncta, which are sites where ER-phagy/nucleophagy occurs.**

3. In Figure S1E, the number of double ring structures in cells treated with DTT is larger than that in nitrogen starved cells. This seems inconsistent with the results of the GFP cleavage assay (Figure 1F, G). The authors should mention this apparent discrepancy.

**Response:** **It has been shown by Schuck et al. (Fig. 4A in PMID: 25052096) that** **DTT treatment inhibits vacuolar proteolysis in budding yeast. It is possible that DTT treatment may have a similar effect in fission yeast. If so, the GFP cleavage assay could underestimate the autophagic flux under DTT treatment conditions. In the revised manuscript, we have mentioned the discrepancy and added this potential explanation for the discrepancy.**

4. Based on the results of electron microscopic analysis, the authors claimed that there is no isolation membrane around nucleus- or ER-derived structures. The authors should also confirm this with fluorescence microscopy analysis (colocalization of Atg8 and cytoplasmic Bqt4 (or Pus1) puncta in yep1Δ cells after 12 h DTT treatment.)

**Response: During the revision, we have performed the experiments suggested by the reviewer. The results are presented in Figures S2C and S2D in the revised manuscript. Only approximately 3% of the Bqt4 puncta and Pus1 puncta colocalized with Atg8 puncta in *yep1Δ* cells after 12 h DTT treatment.**

5. The authors should check the levels of proteins expressed from plasmids in Figs. 3K, M, S3F, S5A, 4C, E, G, H, S6D, E, G, H.

**Response: We have followed the suggestion of the reviewer and added data showing the levels of proteins expressed from plasmids in the revised manuscript. The new data are presented** **in Figures 3K, 3L, S4F, S6A, 4C, 4F, 4H, 4I, S7D, S7E, S7G, and S7H in the revised manuscript.**

6. In Figure S4, some labels are missing.

**Response: We have added the missing labels.**

Reviewer #4: The degradation of ER by autophagy is termed ER-phagy. In order to identify novel factors required for ER-phagy in fission yeast, the authors performed a chemical mutagenesis screen. They screened for mutants which failed to transport the ER membrane protein Ost4-GFP to the vacuole. From this screen they identified a previously uncharacterized protein which they called Yep1. The authors confirm that deletion of yep1 in S. pombe leads to a loss of ER-phagy in response to both DTT and -N. They further demonstrate that yep1 is required for nucleophagy (autophagy of the nucleus) but it is not required for bulk (non-selective) autophagy. Interestingly, the authors demonstrate that Yep1 does not function as a selective autophagy receptor as it can not interact with Atg8 and that it likely contains a different function. EM imaging suggested that stalled ER-phagy cargos in yep1delta cells are not enclosed in autophagosomes. To verify this they use an auxin inducible degradation system to demonstrate that ER-phagy cargos are accessible to the cytoplasm and degraded in -N media. They next demonstrate that deletion of yep1 alters ER morphology suggesting that yep1 plays a role in ER shaping. They demonstrate that yep1 can oligomerize (likely a dimer) by co-IP and BIFC. They demonstrate that Yep1 shares similar features with the REEP family of proteins and Atg40 (an ER-phagy receptor in S. cerevisiae). They demonstrate that REEP1-4 and Atg40 can rescue the ER-phagy defect of yep1 suggesting that these proteins have similar roles in ER-phagy. Taken together all of their data suggests one of two possible roles for yep1 in ER-phagy and nucleophagy. The first is that yep1 plays a role in shaping cargo membranes while in the second it may play a role in shaping the autophagosomal membrane.

This is a very interesting paper with new and exciting findings about the mechanisms of autophagy. The paper is well written and the conclusions are well supported. The authors were careful to use complimentary assays (co-IP and BIFC or light microscopy and EM) to strengthen their findings. They authors also make good use of controls for the majority of their assays. It is also nice to see the honesty and openness of the discussion section. The authors are careful to state different possibilities for their data. They also carefully compare their data to another recent manuscript without criticizing the other paper. One major weakness of the manuscript is the lack of quantification of their ER-phagy, nucleophagy and bulk autophagy assays. The authors have many instances where a single western blot is shown for each experiment. Repeating these assays in triplicate and quantification of these blots would further strengthen their findings.

Major concerns

1. The authors rely heavily on ER-phagy, nucleophagy and bulk autophagy assays which show the generation of free GFP from a protein that is captured in these processes. However, this results in individual western blots where only single points of data are shown. The authors should repeat these results in triplicate for all of these experiments in the main figures and quantify them to strengthen their claims. The authors should repeat and quantify Figure 1D, 1E, 1F, 1G, 1H, 1I, 1J, 3J, 3K, 3M 4C, 4E, 4G, 4H.

**Response: We thank the reviewer for the positive evaluation of our manuscript and for offering valuable suggestions. We have repeated these western blots and quantified the cleavage ratios as suggested by the reviewer. The quantification results are presented in Figures 1D, 1E, 1F, 1G, 1H, 1I, 1J, 3J, 3K, 3L 4C, 4F, 4H, 4I in** **the revised manuscript.**

2. It is not entirely clear how the authors noted the formation of the Bqt4 and Pus1 puncta that are utilized in Figure 2. It would be helpful if this could be explained in more detail in the manuscript.

**Response: We used the processing of Pus1-mECitrine as a readout of nucleophagy. Microscopic analysis of cells expressing Pus1-mECitrine led to the observation that Pus1-mECitrine formed cytoplasmic puncta in *yep1Δ* cells under conditions inducing ER-phagy/nucleophagy. Subsequently, we analyzed cells expressing Bqt4-mECitrine and found that it also formed cytoplasmic puncta *yep1Δ* cells under conditions inducing ER-phagy/nucleophagy.** **We added more detailed description of how we noticed these phenotypes in the revised manuscript.**

3. In the CO-IP experiments in Figure 3D and 3H, why does Yep1-GFP appear as a doublet? If the 34 kDa band is largely free GFP or mcherry why does this pulldown with the sample? Do the authors have a hypothesis for this doublet.

**Response: Based on the apparent molecular weights, the top band of the doublet likely corresponds to full-length Yep1-GFP/Yep1-mCherry and the lower band of the doublet likely corresponds to an N-terminal cleaved form of Yep1-GFP/Yep1-mCherry. We performed further experiments to assess where this cleavage occurs. We hypothesized the cleavage likely occurs in the regions between transmembrane helices (TMHs), and therefore expressed N-terminally truncated Yep1 that begins immediately after the first inter-TMH region or after the second inter-TMH region. We found that Yep1(35-166)-mCherry, which begins after the first inter-TMH region, still appeared as a doublet****, whereas Yep1(79-166)-mCherry, which begins after the second inter-TMH region, appeared as a single band migrating at a position slightly lower than the bottom band of the doublet. These results indicate that the cleavage probably occurs between the second and the third TMHs. Interestingly, unlike the bottom band of the doublet of Yep1-mCherry, which can be co-immunoprecipitated by Yep1-GFP, neither Yep1(35-166)-mCherry nor Yep1(79-166)-mCherry was co-immunoprecipitated by Yep1-GFP, indicating that the N-terminal region of Yep1 is necessary for self-interaction. Our interpretation of these results is that the cleavage, which likely happened during protein extraction, did not cause the dissociation of the N-terminal region from the rest of the molecule under non-denaturing immunoprecipitation conditions and also did not affect the self-interaction. We present these new results in Figures S5F-S5H** **in the revised manuscript.**

4. In Figure 3F the overall structure is hard to compare to 3G. It may be helpful to color one of the monomers in 3F grey and the other monomer the colors used in 3G. This would help connect the two figures and make it clear which helices are the APHs to be discussed later.

**Response: We have followed the reviewer’s suggestion and changed the color of Figure 3F in the revised manuscript.**

5. The authors mention that mutation of the APH1 helix did not abolish ER-phagy but it did lead to a dramatic reduction in ER-phagy in Figure 3M. The authors should acknowledge this in the results.

**Response: We have revised the manuscript as suggested by the reviewer.**
